# Remodeling the Proteostasis Network to Rescue Glucocerebrosidase Variants by Inhibiting ER-Associated Degradation and Enhancing ER Folding

**DOI:** 10.1371/journal.pone.0061418

**Published:** 2013-04-19

**Authors:** Fan Wang, Laura Segatori

**Affiliations:** 1 Department of Chemical and Biomolecular Engineering, Rice University, Houston, Texas, United States of America; 2 Department of Biochemistry and Cell Biology, Rice University, Houston, Texas, United States of America; 3 Department of Bioengineering, Rice University, Houston, Texas, United States of America; University Hospital S. Maria della Misericordia, Udine, Italy

## Abstract

Gaucher’s disease (GD) is characterized by loss of lysosomal glucocerebrosidase (GC) activity. Mutations in the gene encoding GC destabilize the protein’s native folding leading to ER-associated degradation (ERAD) of the misfolded enzyme. Enhancing the cellular folding capacity by remodeling the proteostasis network promotes native folding and lysosomal activity of mutated GC variants. However, proteostasis modulators reported so far, including ERAD inhibitors, trigger cellular stress and lead to induction of apoptosis. We show herein that lacidipine, an L-type Ca^2+^ channel blocker that also inhibits ryanodine receptors on the ER membrane, enhances folding, trafficking and lysosomal activity of the most severely destabilized GC variant achieved via ERAD inhibition in fibroblasts derived from patients with GD. Interestingly, reprogramming the proteostasis network by combining modulation of Ca^2+^ homeostasis and ERAD inhibition remodels the unfolded protein response and dramatically lowers apoptosis induction typically associated with ERAD inhibition.

## Introduction

Lysosomal storage disorders (LSD) are a group of more than 40 clinically distinct inherited diseases characterized by the deficiency of essential lysosomal hydrolytic functions [1]. Gaucher’s disease (GD), the most common LSD, is caused by loss of lysosomal glucocerebrosidase (GC) activity and consequent accumulation of the GC substrate, glucosylceramide [2].

The most frequently encountered mutations in the GC encoding gene (*GBA*; [3]) do not directly affect the enzyme’s activity but destabilize its native structure [4]. They are typically single amino acid substitutions that impair the enzyme’s folding causing retro-translocation of misfolded GC to the cytoplasm for ER-associated degradation (ERAD) and, consequently, leading to deficiency of lysosomal GC activity [5]. The L444P substitution is one of the most frequently occurring misfolding mutations [4]. It severely destabilizes GC native structure and results in complete loss of activity [6]. GD patients who are homozygous for the L444P GC allele typically present severe neuropathic symptoms [6].

Interestingly, a number of unstable GC variants containing misfolding mutations (including L444P GC) can traffic to the lysosome and retain catalytic function if forced to fold into their native 3D structure [7]–[10]. Chemical chaperones, small molecules that rescue the native folding of mutated GC enabling lysosomal trafficking and enhancing enzyme activity were recently reported [8]. However, chemical chaperones are highly mutation-specific [11] and rarely proved effective to rescue GC variants associated with neuropathic manifestations of the disease [12]. Modulation of the proteostasis network has been explored recently to restore the activity of GC variants in cells derived from GD patients with neuropathic symptoms [10], [13]–[15]. The ultimate goal of this approach is to achieve chemically induced enhancement of the innate cellular folding capacity – a strategy that could be in principle applicable to rescue the function of a large class of mutated enzymes processed through the secretory pathway [10]. However, the mechanism of action of most small molecules reported to function as proteostasis modulators thus far relies on induction of cellular stress, and, particularly, activation of the unfolded protein response (UPR). Sustained UPR activation, in turn, leads to induction of apoptosis [13], [14]; hence the recent focus on modulating the cellular folding capacity to rescue the folding of unstable, degradation-prone proteins without causing induction of the apoptotic cascade [13], [14].

Native folding of GC variants is limited by rapid disposal of unstable folding intermediates via ERAD [5]. We previously reported that chemical inhibition of specific steps of the ERAD pathway enables rescue of folding and trafficking of mutated GC [15]. Particularly, administration of Eeryastatin I (EerI), a small molecule that blocks retrotranslocation of misfolded substrates to the cytoplasm [16], [17], resulted in dramatic rescue of folding and lysosomal activity of multiple GC variants. ERAD inhibition via EerI treatment was shown to prolong ER retention of mutated GC, thereby enhancing the pool of GC folding intermediates amenable to folding rescue. However, EerI treatment was also observed to cause dramatic induction of UPR and apoptosis [15].

Accumulation of the GC substrate (glucosylceramide) in GD cells causes excessive [Ca^2+^]_ER_ efflux through the ryanodine receptors (RyRs) [18]–[20]. Because maintenance of intracellular Ca^2+^ homeostasis is essential for a number of fundamental cellular activities including protein folding in the ER [21], [22], impairment of intracellular Ca^2+^ homeostasis in GD cells is likely to hamper the folding of unstable GC variants [13], [23]. Re-establishing the cellular gradient of [Ca^2+^] in GD fibroblasts was shown to create an ER environment more amenable to native folding of GC variants [13], [14]. Lowered [Ca^2+^] in the cytosol observed upon treatment of GD cells with lacidipine, a small molecule that inhibits both RyRs on the ER membrane and L-type Ca^2+^ channels (LTCC) on the plasma membrane [24], [25], correlates with the increase in trafficking and lysosomal activity of L444P GC [14]. Interestingly, despite causing moderate activation of the UPR, lacidipine treatment was observed to prevent apoptosis induction, effectively promoting cell survival [14].

**Figure 1:**
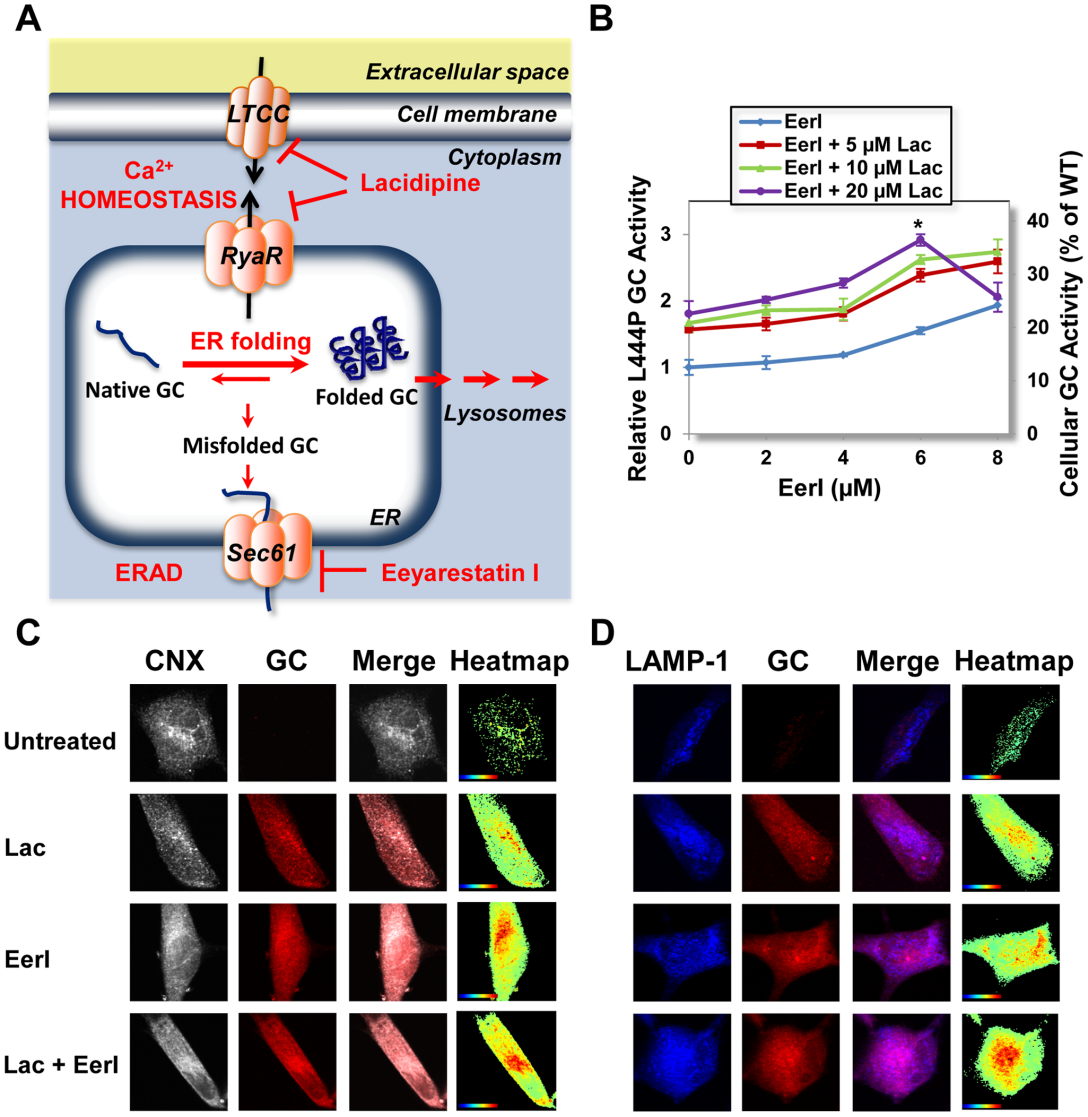
Co-treatment with EerI and lacidipine enhances lysosomal trafficking and activity of L444P GC. (**A**) Lacidipine and EerI modulate distinct pathways of the proteostasis network that regulate the processing of GC. Lacidipine enhances ER folding by restoring Ca^2+^ homeostasis in GD cells [14]. Specifically, lacidipine inhibits extracellular Ca^2+^ influx through L-type voltage-gated Ca^2+^ channels (LTCC) on the plasma membrane and blocks ER Ca^2+^ efflux through ryanodine receptors (RyRs) on the ER membrane, thus restoring the intracellular gradient of [Ca^2+^]. EerI treatment enhances retention of unstable proteins in the ER. Particularly, EerI inhibits p97 ATPase activity, which promotes retro-translocation of misfolded substrates from the ER to the cytoplasm for ER-associated degradation (ERAD). (**B**) L444P GC activities of GD cells treated with a range of concentrations of EerI and constant doses of lacidipine (5, 10, or 20 µM) for 48 hrs. Relative GC activities were evaluated by normalizing GC activities measured in treated cells to the activity in untreated cells (left y axis), (ANOVA, p<0.01 if not specified; *p<0.001). The corresponding fraction of WT GC activity is also reported (right y axis). Experiments were repeated three times and data points are reported as mean ± SD. Lac, lacidipine. (**C–D**) Immunofluorescence microscopy of GC and CNX (an ER marker), and GC and LAMP-1 (a lysosomal marker) in L444P GC fibroblasts. Cells were treated with EerI (6 µM), and lacidipine (10 µM) for 48 hrs. (**C**) Colocalization of CNX (grey, column 1) and GC (red, column 2) is shown in pink (column 3). (**D**) Colocalization of LAMP-1 (blue, column 1) and GC (red, column 2) is shown in purple (column 3). Heatmaps of co-localization images were obtained with NIH ImageJ analysis software (column 4). Hot colors represent positive correlation (co-localization), whereas cold colors represent negative correlation (exclusion).

We hypothesized that restoring Ca^2+^ homeostasis in GD cells creates a folding environment that could be particularly amenable to enhance native folding and trafficking of mutated GC mediated by ERAD inhibition. Thus, we attempted remodeling the proteostasis network by simultaneously i) inhibiting ERAD degradation to increase ER retention of unstable intermediates and ii) restoring Ca^2+^ homeostasis to enhance chaperone-mediated folding (Figure 1A). We report herein that this strategy results in dramatic increase in the folding, trafficking and activity of the most severely destabilized GC variant, L444P GC. Moreover, we demonstrated that modulation of Ca^2+^ homeostasis via lacidipine treatment lowers UPR induction and apoptosis caused by ERAD inhibition. Results from this study provide novel insights for the development of effective therapeutic strategies for the treatment of GD based on remodeling the proteostasis network to rescue the folding of unstable, degradation-prone GC variants.

## Materials and Methods

### Enzyme Activity Assays

The intact cell glucocerebrosidase (GC) activity assay was performed as described previously [10] and in File S1.

### Quantitative RT-PCR

Quantitative RT-PCR was performed as described previously [15] and in Materials S1 using the primers listed in Table S1.

### Western Blot Analyses and Immunofluorescence Microscopy

Details are provided in File S1.

### siRNA Transfection

Transfection procedures were performed as described in manufacturer’s manual: briefly, 12.5 ng siRNA was diluted in 3 µl of RNase-free water and was spotted into each well of a 96-well plate. 0.75 µl of HiPerFect reagent was resuspended in 25 µl of culture medium without serum and was added to the prespotted siRNA. After incubating for 10 min at room temperature to allow complex formation, 10^4^ cells in 150 µl of growth medium were plated directly into each well. Small molecules were added to the medium 48 hrs post transfection at concentrations indicated in the text. Quantitative RT-PCR was performed after 24 hrs and lysosomal GC activity was measured after 48 hrs.

### Toxicity Assay

Toxicity assays were conducted as described previously [15] and in File S1.

### Statistical Analysis

All data is presented as mean ± s.d., and statistical significance was calculated using one-way ANOVA analysis followed by post-hoc Tukey’s test.

## Results

### Modulation of Ca^2+^ Homeostasis Enhances the Rescue of L444P GC Folding, Trafficking and Activity Induced by ERAD Inhibition in GD Fibroblasts

We previously reported that the folding of mutated GC variants is partially rescued by inhibiting specific steps of the ERAD pathway in GD cells [15]. In the present study, we asked whether enhancing the cellular folding capacity via modulation of intracellular Ca^2+^ homeostasis could increase the fraction of natively folded GC mutants rescued by inhibiting ERAD (Figure 1A) [13], [14]. We co-administered the LTCC blocker lacidipine, which lowers cytosolic [Ca^2+^] in GD fibroblasts [14], and Eeyarestatin I (EerI), which blocks the ERAD pathway by inhibiting the p97 ATPase [16], [17], to fibroblasts derived from GD patients homozygous for the L444P GC allele and investigated the activity and intracellular trafficking of mutated GC. Experiments were performed by administrating a constant concentration of lacidipine (5, 10, or 20 µM) to GD fibroblasts that were cultured in medium supplemented with a range of EerI concentrations. GC enzymatic activity was evaluated every 24 hrs for up to 72 hrs with the intact cell GC activity assay (Figures 1B and S1). Culturing conditions resulting in maximal rescue of L444P GC activity are reported in Figure 1B. Co-treatment with EerI (6 µM) and lacidipine (20 µM) for 48 hrs resulted in 2.9-fold increase in L444P GC activity compared to untreated cells (ANOVA, p<0.001; F = 16; Figure 1B), which corresponds to 36.3% of WT activity and is compatible with effective treatment [2]. This increase in GC activity is significantly higher (p<0.001) than that measured in cells treated only with EerI (1.6-fold; ANOVA, p<0.01, F = 22) or lacidipine (1.8-fold; ANOVA, p<0.01, F = 16) under the same conditions and was still observed after 72 hrs of incubation (EerI 6 µM and lacidipine 20 µM, 2.6-fold increase in GC activity; ANOVA, p<0.01, F = 14; Figure S1).

In order to verify that the increase in GC activity observed in cells treated with EerI and lacidipine is due to rescue of L444P GC folding and lysosomal trafficking, we investigated L444P GC intracellular localization. Cells were treated to obtain maximal increase in GC activity and analyzed by immunofluorescence microscopy. Specifically, L444P GC fibroblasts were cultured with EerI (6 µM), lacidipine (10 µM) and a combination thereof for 48 hrs. Localization of GC in the ER and in the lysosomes was detected with antibodies specific for GC, for an ER marker (CNX), and for a lysosomal marker (LAMP-1). Co-localization of GC and CNX (Figure 1C) and of GC and LAMP-1 (Figure 1D) are shown in pink and purple, respectively, in merged images. As shown in heatmaps of co-localization images, L444P GC was barely detectable in untreated cells due to extensive ERAD, as expected [10]. Treatment with lacidipine or EerI enhanced the pool of GC that accumulates both in the ER and in the lysosomes, as previously reported [14], [15]. The addition of lacidipine to EerI treatment resulted in accumulation of GC in the ER similar to that observed in cells treated only with EerI. However, co-treatment with lacidipine and EerI resulted in an increase in accumulation of GC in the lysosomes compared to cells treated with either EerI or lacidipine (Figure 1CD). These results are compatible with a model in which combining modulation of Ca^2+^ homeostasis and ERAD inhibition enhances rescue of GC folding intermediates that escape ERAD and promotes their trafficking through the secretory pathway, thereby leading to the increase in lysosomal GC activity observed from enzymatic assays (Figure 1B).

### Lacidipine Lowers Cytosolic Free [Ca^2+^] in GD Fibroblasts Treated with EerI

**Figure 2:**
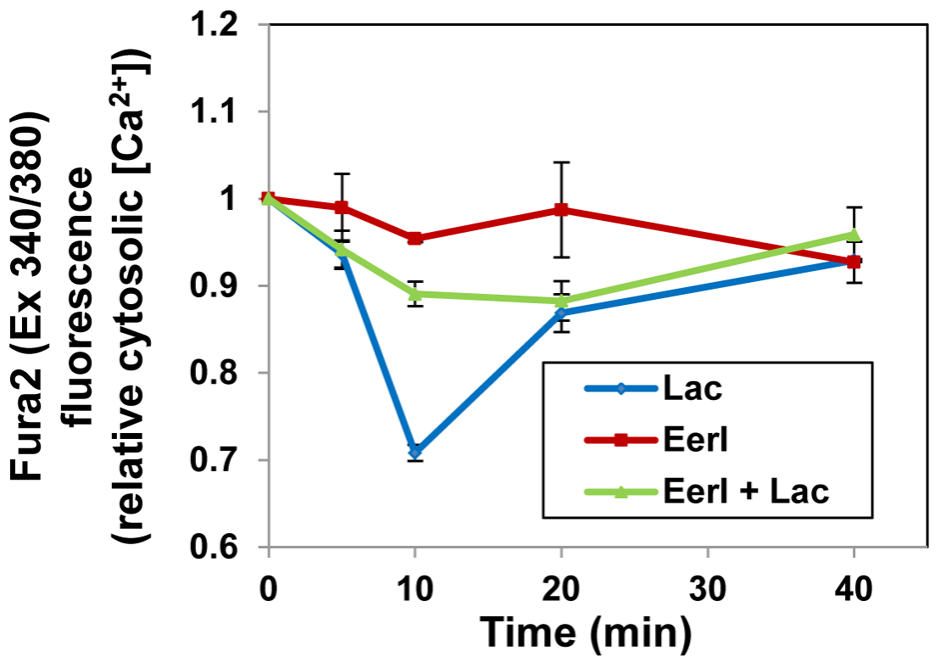
Lacidipine reduces cytosolic [Ca^2+^] in GD fibroblasts treated with EerI. GD fibroblasts were cultured with lacidipine (10 µM) and EerI (6 µM) for 5, 10, 20 and 40 min, respectively. Cytosolic [Ca^2+^] level was evaluated by measuring excitation 340/380 ratio of fura-2 acetoxymethyl ester and normalized to that at time zero. Each data point was repeated three times and reported as mean ± SD.

The accumulation of glucosylceramide in GD cells causes Ca^2+^ efflux from the ER and increases free cytosolic [Ca^2+^] [18]. We previously showed that lacidipine treatment lowers cytosolic [Ca^2+^] in GD fibroblasts and, in turn, is associated with an increase in mutated GC folding and activity. Since administration of lacidipine to EerI-treated cells increases the residual activity of L444P GC (Figure 1B), we asked whether this difference in activity could be attributed to the mobilization of intracellular Ca^2+^. We evaluated cytosolic free [Ca^2+^] in L444P GC fibroblasts treated with EerI (6 µM), lacidipine (10 µM) and a combination thereof by measuring fluctuations in the Fura-2 fluorescence ratio (340 nm/380 nm) [23]. EerI treatment alone did not seem to alter intracellular [Ca^2+^] in GD fibroblasts. However, the addition of lacidipine to EerI-treated cells lowered free cytosolic [Ca^2+^] (Figure 2). Interestingly, the free cytosolic [Ca^2+^] in GD cells treated with lacidipine was lower than that observed in cells co-treated with lacidipine and EerI, suggesting that either minimal intracellular Ca^2+^ mobilization is sufficient to enhance L444P GC folding and activity or that the increase in L444P GC activity obtained upon co-administration of lacidipine and EerI is due to additional or alternative effects of these compounds on the proteostasis network.

### Lacidipine Attenuates the Cytotoxic Effect of EerI-mediated ERAD Inhibition in GD Fibroblasts

EerI treatment causes accumulation of misfolded intermediates in the ER and, consequently, ER stress and induction of the UPR [15]. Moderate UPR induction was repeatedly reported to promote the rescue of misfolding-prone GC variants [10], [13]–[15]. However, prolonged UPR induction observed upon sustained treatment with EerI causes activation of apoptosis [14], [15]. Cell treatment with lacidipine, on the other hand, was shown not to cause cytotoxicity under conditions observed to rescue the folding of mutated GC variants [14].

**Figure 3:**
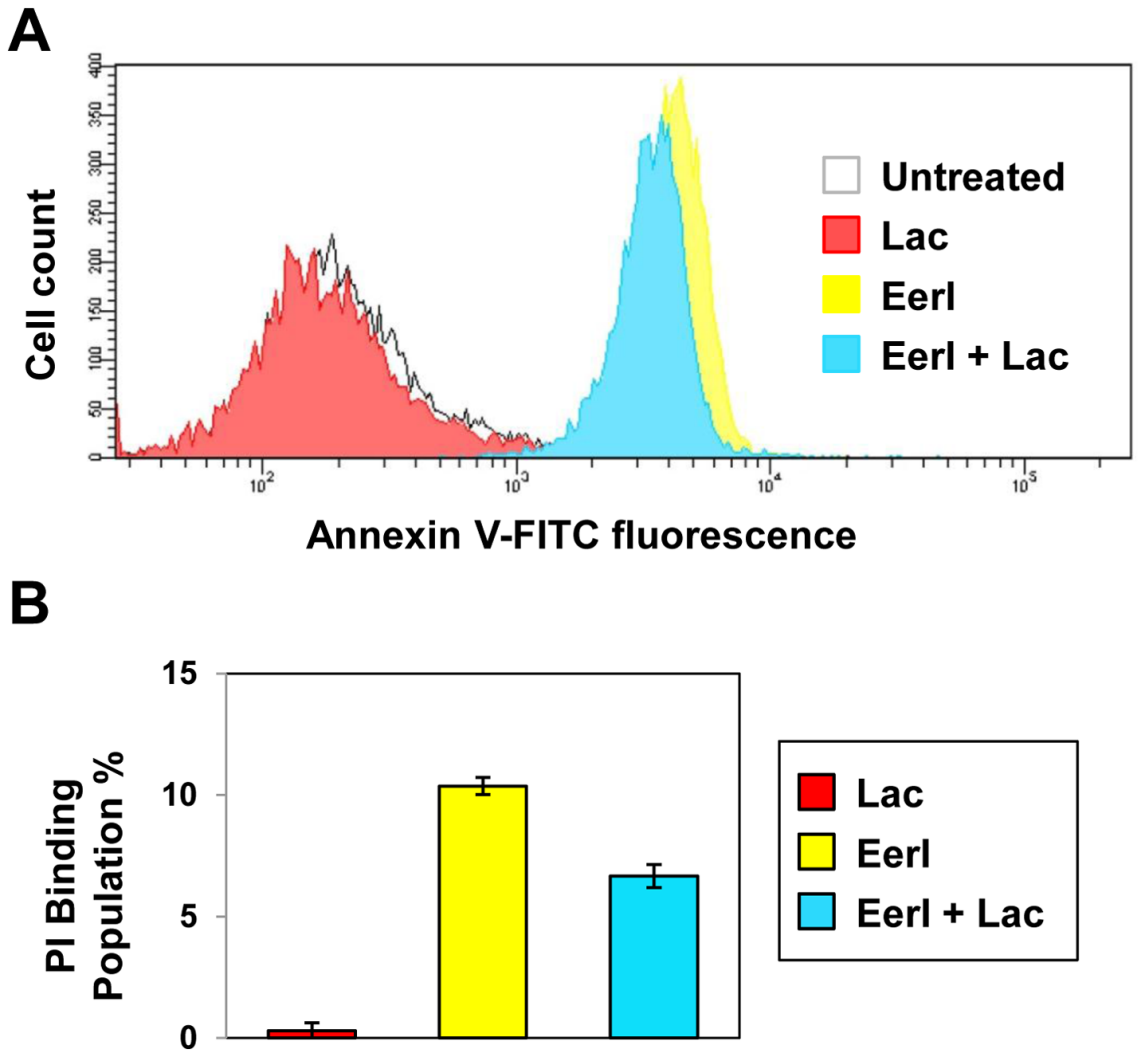
Lacidipine attenuates induction of apoptosis in GD cells treated with EerI. (**A**) Flow cytometry histograms of Annexin V-FITC fluorescence intensities (x-axis, log scale) plotted against cell counts (y-axis, linear scale) obtained from the analysis of untreated cells and cells treated with lacidipine (10 µM) and EerI (6 µM). Three independent experiments were conducted and results of one representative experiment are reported. (**B**) PI binding population change (%) of cells treated with lacidipine (10 µM) and EerI (6 µM) for 16 hrs compared to untreated cells (ANOVA, p<0.01). Number of total cells counted: 10,000. The data is reported as mean ± SD.

We asked whether lacidipine treatment could counteract the cytotoxic effect of EerI and evaluated apoptosis in cells co-treated with lacidipine and EerI. Specifically, we monitored membrane rearrangement (Annexin V binding) and fragmentation (propidium iodide (PI) binding) that occur during early and late apoptosis, respectively, using the CytoGLO™ Annexin V-FITC Apoptosis Detection Kit. L444P GC fibroblasts were cultured with lacidipine (10 µM) and EerI (6 µM) for 16 hrs (Figure 3A–B). Similar to what previously reported [14], [15], Annexin V binding affinity in cells treated with lacidipine was comparable to that measured in untreated cells, whereas a dramatic increase in Annexin V binding was observed in cells treated with EerI, reflecting the onset of apoptosis. The addition of lacidipine to EerI-treated cells resulted in significant decrease in Annexin V binding compared to cells treated only with EerI (14% decrease; ANOVA, p<0.01), suggesting that lacidipine treatment partially alleviates the cytotoxic effect of EerI (Figure 3A). Cell death was measured by monitoring the change in PI binding population. A negligible increase (0.3%) in PI binding was observed upon lacidipine treatment, while EerI treatment caused 10.4% increase in PI binding (ANOVA, p<0.01; Figure 3B). The addition of lacidipine to EerI-treated cells reduced the PI binding population to 6.7% (ANOVA, p<0.01), confirming that lacidipine treatment has an anti-apoptotic effect.

These results, taken together, demonstrate that lacidipine treatment enhances EerI-mediated rescue of mutated GC native folding and activity and that it also counteracts EerI cytotoxic effect. Moreover, lacidipine treatment protects the cells from apoptosis associated with prolonged UPR induction, a particularly appealing property for development of therapeutic strategies based on the modulation of the proteostasis network.

### Lacidipine Remodels EerI-mediated Activation of the UPR Pathway

EerI, when administered under conditions that result in maximal increase in L444P GC activity, is associated with significant UPR induction and cell apoptosis, whereas lacidipine treatment induces UPR but does not cause apoptosis [14], [15]. We reported above that co-administration of lacidipine and EerI lowers apoptosis compared to treatment with EerI only (Figure 3). Therefore, we asked whether co-treatment of GD cells with EerI and lacidipine activates the UPR. The UPR is a complex tripartite pathway regulated by three transmembrane signal transducers, namely inositol requiring kinase 1 (IRE1), activating transcription factor 6 (ATF6) and double-stranded RNA-activated ER kinase (PERK). Activation of these three sensors leads to transcriptional regulation of a series of UPR target genes that mediate cellular folding [26], [27]. In order to evaluate UPR induction, we measured the expression of three representative UPR target proteins: X-box binding protein-1 (Xbp-1), which is activated by IRE1; activating transcription factor 4 (ATF4), which is part of the PERK signaling cascade; and C/EBP homologous protein (CHOP), which is upregulated in response to ATF6 activation [27]. Quantitative RT-PCR was conducted to evaluate the transcription levels of *Xbp-1*, *ATF4*, and *CHOP* in cells treated with lacidipine (10 µM) and EerI (6 µM).

**Figure 4:**
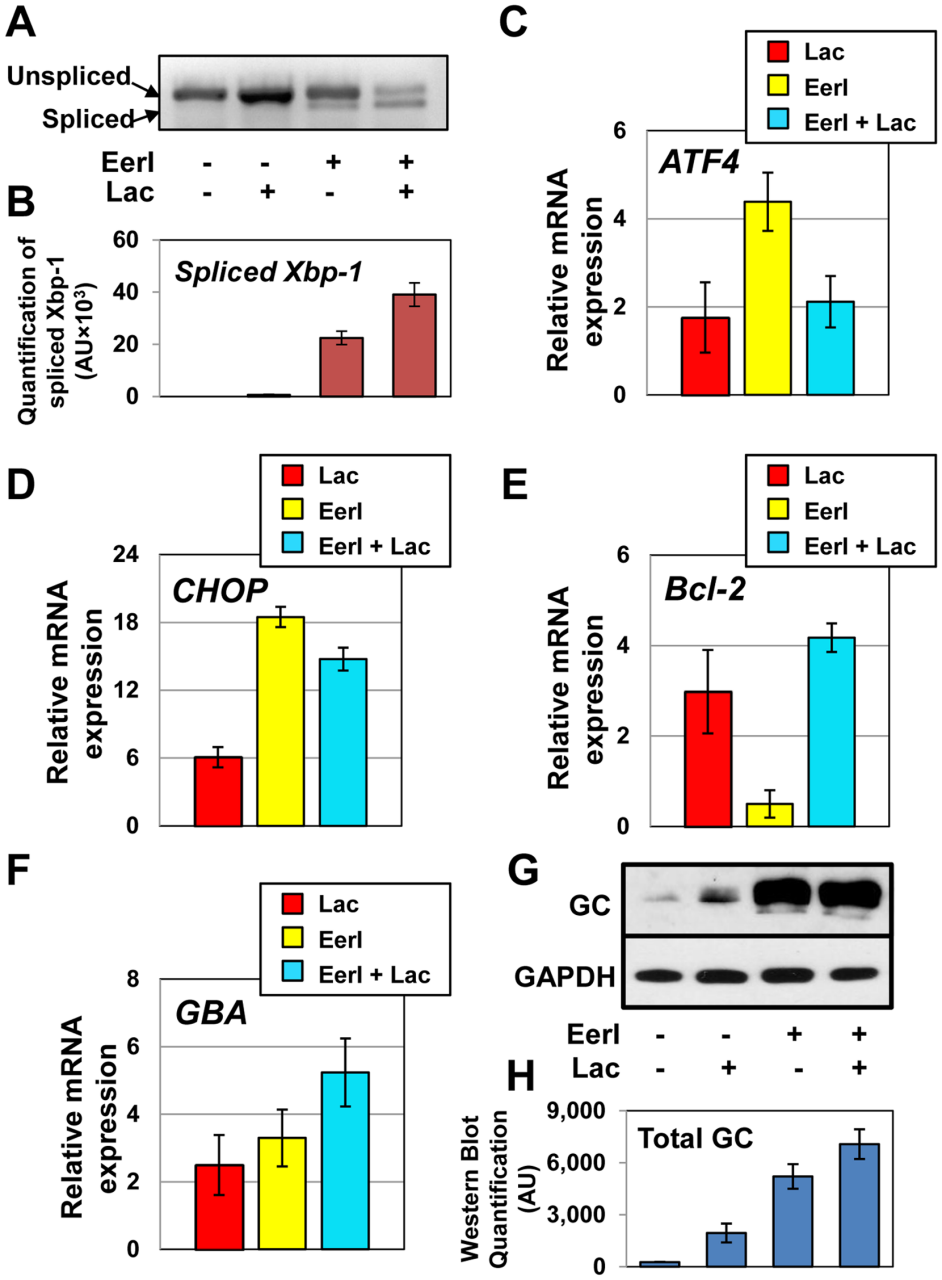
Lacidipine remodels the UPR pathway in GD fibroblasts treated with EerI. Cells were treated with lacidipine (10 µM) and EerI (6 µM) for 24 hrs. (**A**) Xbp-1 mRNA splicing was determined by RT-PCR followed by gel electrophoresis. (**B**) Spliced Xbp-1 band intensities were quantified with NIH ImageJ analysis software. Relative mRNA expression levels of (**C**) ATF4, (**D**) CHOP, (**E**) Bcl-2, and (**F**) GC were obtained by quantitative RT-PCR, corrected by the expression of the housekeeping gene GAPDH, and normalized by that of untreated cells (ANOVA, p<0.05). The data is reported as mean ± SD. (**G**) Western blot analysis of cells treated with EerI (6 µM) and lacidipine (10 µM) for 48 hrs using GC specific antibody. GAPDH expression was used as a loading control. (**H**) Western blot band quantification. GC bands were quantified by NIH ImageJ analysis software and corrected by GAPDH band intensities.

*Xbp-1* mRNA is spliced upon activation of the IRE1 signaling cascade. The protein encoded by the spliced *Xbp-1* mRNA functions as an activator of the IRE1 branch of the UPR, while the protein encoded by the unspliced precursor acts as a repressor [26]. Spliced and unspliced forms of *Xbp-1* mRNA were analyzed by RT-PCR followed by gel electrophoresis. Bands corresponding to spliced *Xbp-1* mRNA were quantified with NIH ImageJ software to evaluate the activation level of the IRE1 arm of the UPR (Figure 4A–B). *Xbp-1* splicing in cells treated with lacidipine or with EerI was previously investigated [14], [15]. Thus, EerI- and lacidipine-treated cells are reported here as control samples. In agreement with what reported before [14], the amount of spliced *Xbp-1* in lacidipine treated cells was similar to that measured in untreated cells, while a considerable amount of spliced *Xbp-1* was observed in cells treated with EerI [15]. In cells treated with both lacidipine and EerI, the amount of spliced *Xbp-1* was found to increase 1.8-fold compared to cells treated only with EerI, suggesting an additive effect of lacidipine and EerI on the induction of the IRE1 arm. Interestingly, Xbp-1 is an essential pro-survival UPR component and its activation is associated with attenuated apoptosis under ER stress conditions [28]. Thus, enhanced splicing of *Xbp-1* in cells treated with lacidipine and EerI correlates with the decrease in apoptosis induction observed in cells treated under same conditions.

The expression level of *ATF4* was evaluated in order to monitor the activation of the PERK branch. *ATF4* transcriptional expression was upregulated 1.8- and 4.4-fold in cells treated with lacidipine and EerI, respectively, compared to untreated cells. Co-treatment with lacidipine and EerI reduced *ATF4* expression to only 2.1-fold of that of untreated cells (ANOVA, p<0.05), suggesting that lacidipine suppresses EerI-mediated activation of the PERK arm (Figure 4C). Since the PERK arm of the UPR regulates the pro-apoptotic pathway activated in response to sustained UPR induction [29], these results support the notion that lacidipine lowers the apoptotic effect of EerI.

CHOP, a downstream effector of the ATF6 branch, is upregulated by both lacidipine and EerI treatment (6.1- and 18.5-fold, respectively; Figure 4D). The addition of lacidipine to EerI-treated cells lowered *CHOP* upregulation to 14.7-fold (ANOVA, p<0.05). CHOP mediates UPR induced apoptosis activation [30]. Hence, these results again suggest a correlation between lacidipine’s anti-apoptotic effect and its ability to remodel the UPR pathway activated by EerI.

Lacidipine treatment alters the expression of genes involved in the regulation of UPR-induced apoptosis, and, particularly, it upregulates the anti-apoptotic gene *Bcl-2*. We therefore asked whether the protective effect of lacidipine treatment observed in EerI-treated cells could be attributed to the upregulation of *Bcl-2* [14]. The expression level of *Bcl-2* was evaluated by quantitative RT-PCR in cells cultured with lacidipine (10 µM) and EerI (6 µM). Lacidipine treatment resulted in 3.0-fold increase in *Bcl-2* expression compared to untreated cells, while EerI treatment caused a 2.0-fold decrease. *Bcl-2* expression was also significantly upregulated (4.2-fold; ANOVA, p<0.05) in cells co-treated with lacidipine and EerI (Figure 4E).

The gene encoding GC (*GBA*), as well as other genes encoding for lysosomal proteins that are associated with the development of LSD, is upregulated in cells treated with proteostasis modulators [14], [15]. We thus asked whether the dramatic increase in L444P GC activity observed upon modulation of intracellular Ca^2+^ homeostasis and inhibition of ERAD could be attributed to upregulation of GC transcription in addition to rescue of GC folding and inhibition of GC degradation. The expression of *GBA* in GD fibroblasts treated with lacidipine (10 µM) and EerI (6 µM) was measured by quantitative RT-PCR. Co-administration of lacidipine and EerI resulted in 5.2-fold upregulation of GC expression compared to untreated cells (ANOVA, p<0.05), which is higher than what observed in cells treated only with lacidipine (2.5-fold) or EerI (3.3-fold) (Figure 4F).

GC expression was also evaluated by Western blot (Figure 4G–H). As shown in Figure 4H, L444P GC content was barely detectable in untreated cells, as expected, due to extensive ERAD [8], but it was enhanced by treatment with either lacidipine or EerI. In agreement with the results obtained from quantitative RT-PCR analyses, co-treatment with lacidipine and EerI further enhanced GC accumulation (1.4-fold increase compared to EerI treatment alone).

**Figure 5:**
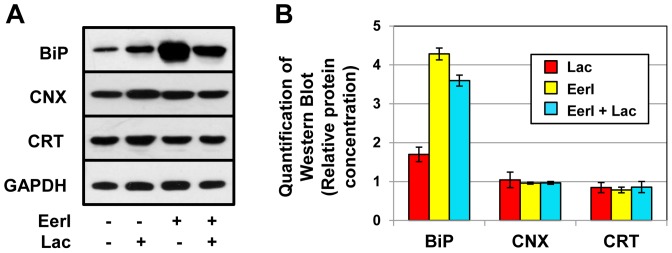
Lacidipine attenuates BiP upregulation in GD cells treated with EerI. (**A**) Western blot analyses of BiP, CNX, CRT, and GAPDH (used as loading control) in GD fibroblasts treated with lacidipine (10 µM) and EerI (6 µM) for 48 hrs. (**B**) Quantification of Western blot bands. ER chaperone band intensities were quantified with NIH ImageJ analysis software, corrected by GAPDH band intensities, and divided by the values obtained from untreated samples.

Among ER resident chaperones, BiP plays a critical role in the folding of mutated GC variants [13], [14]. The increase in lysosomal GC activity observed upon chemically induced inhibition of ERAD or modulation of intracellular Ca^2+^ homeostasis in GD cells is partially due to the upregulation of BiP expression associated with UPR induction. Thus, we evaluated BiP expression in cells treated with lacidipine and EerI and compared it to that of other ER chaperones Calnexin (CNX) and Calreticulin (CRT). The total protein content of treated and untreated cells was analyzed by Western blot using a BiP-specific antibody (Figure 5A–B). Co-administration of lacidipine and EerI resulted in 3.6-fold increase in BiP cellular accumulation, which is lower than what observed in cells treated only with EerI (4.3-fold). CNX and CRT protein levels were not significantly altered by co-treatment with lacidipine and EerI. BiP is normally upregulated upon activation of the UPR [27]. Thus, the decrease in BiP expression observed in cells treated with EerI and lacidipine correlates with lacidipine-mediated attenuation of UPR induction.

### Bcl-2 does not Directly Affect Mutated GC Folding but Protects GD Cells from Apoptosis Associated with Modulation of Proteostasis

**Figure 6:**
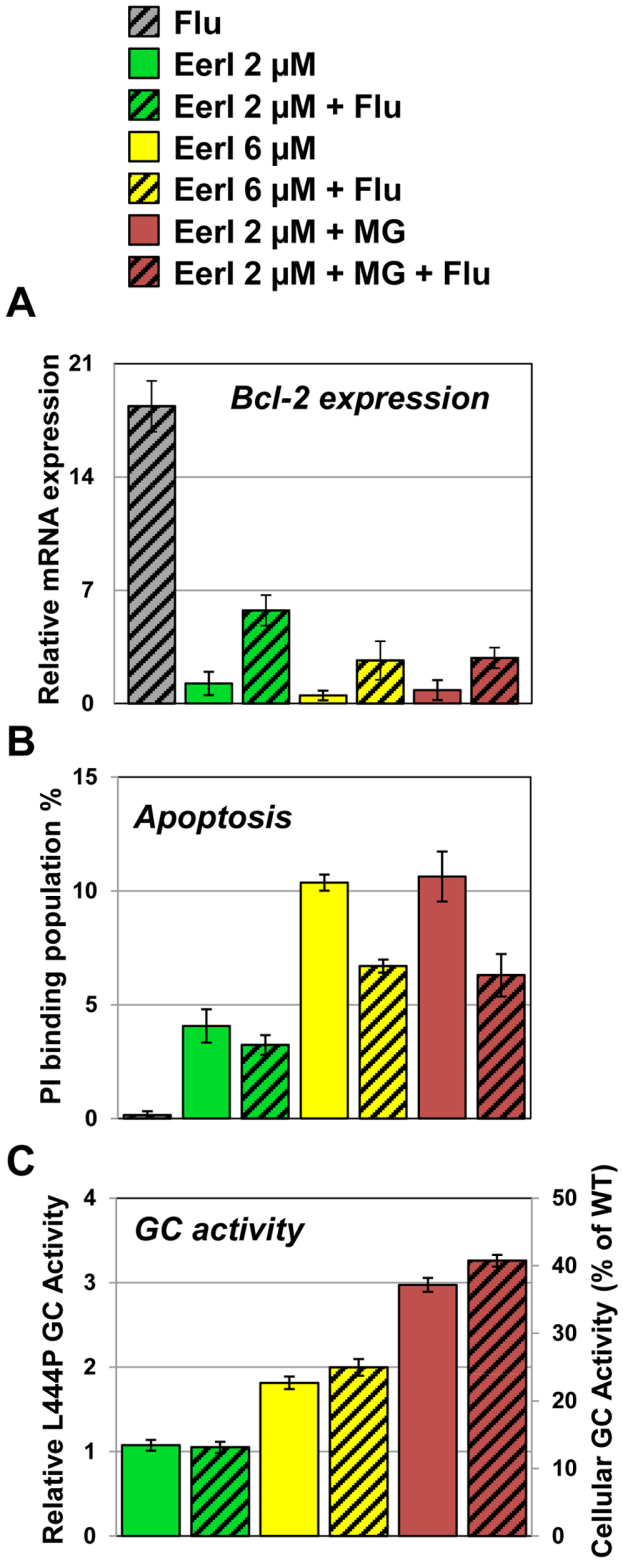
Upregulation of *Bcl-2* protects GD cells from apoptosis induced by proteostasis modulators. (**A**) Relative mRNA expression levels of Bcl-2 in cells treated with EerI (2 and 6 µM), MG-132 (0.6 µM), and fluvastatin (100 nM) for 24 hrs evaluated by quantitative RT-PCR and calculated as described in Figure 4 (ANOVA, p<0.05). (**B**) PI binding population change (%) of cells treated with EerI (2 and 6 µM), MG-132 (0.6 µM), and fluvastatin (100 nM) for 16 hrs compared to untreated cells (p<0.01). The data is reported as mean ± SD. Number of total counted cells: 10,000. (**C**) L444P GC activities of GD fibroblasts treated with EerI (2 and 6 µM), MG-132 (0.6 µM), and fluvastatin (100 nM) for 48 hrs. Relative GC activities were evaluated as described in Figure 1B (ANOVA, p<0.01). Experiments were repeated three times and data points are reported as mean ± SD. MG, MG-132; Flu, fluvastatin.

Bcl-2 is the prototype of an expanding family of proteins that regulate cell survival and apoptosis in multiple cell types [31]. As discussed above, treatment with lacidipine prevents apoptosis associated with UPR induction that was observed upon treatment with EerI. The addition of lacidipine to EerI treated cells results in upregulation of *Bcl-2* expression to a considerably higher level than what is observed administering EerI alone (Figure 4E). In order to investigate the role of Bcl-2 in cells treated for the rescue of mutated GC folding via UPR induction, we treated GD cells with fluvastatin, a compound that was previously reported to induce upregulation of *Bcl-2* [32]. Specifically, we asked whether fluvastatin treatment could counteract the apoptotic effect of prolonged UPR induction. Fluvastatin was administered to GD cells treated with UPR inducing proteostasis modulators known to rescue native folding of mutated GC, namely EerI and MG-132. MG-132 inhibits proteasomal degradation, which, in turn, causes induction of UPR and upregulation of chaperones in GD cells [10]. Co-treatment with EerI and MG-132 was found to dramatically enhance the activity of L444P GC (to 52% of WT activity), but at the cost of even higher induction of apoptosis [15]. We administered fluvastatin (100 nM) to GD cells treated with EerI (2 and 6 µM) and MG-132 (0.6 µM) and tested Bcl-2 expression, induction of apoptosis and GC activity. Fluvastatin treatment caused dramatic upregulation of *Bcl-2* (18.4-fold compared to untreated cells; p<0.05) and did not induce cytotoxicity (Figure 6A–B). The addition of fluvastatin to EerI-treated cells also upregulated *Bcl-2* and lowered apoptosis. Specifically, fluvastatin treatment increased *Bcl-2* expression in cells treated with EerI 2 µM (4.6-fold) and with EerI 6 µM (5.2-fold) (p<0.05; Figure 6A). Fluvastatin treatment also reduced apoptosis caused by EerI, reducing cell death by 40% in cells treated with EerI 6 µM (p<0.01; Figure 6B). Similar results were obtained upon addition of MG-132. Bcl-2 expression was downregulated in cells treated with both EerI and MG-132 (0.8-fold) compared to untreated cells. However, the addition of fluvastatin caused upregulation of *Bcl-2* expression (2.8-fold; ANOVA, p<0.05) compared to untreated cells. The addition of fluvastatin also resulted in a decrease in cell death (40%; ANOVA, p<0.01) compared to cells treated only with EerI and MG-132. These results suggest that Bcl-2 plays a protective role in GD cells treated with proteostasis modulators that induce the UPR and activate apoptosis.

To test whether chemically induced upregulation of Bcl-2 expression affects rescue of L444P GC activity, we also measured GC activity in GD cells cultured under the same conditions (Figures 6C and S2). Interestingly, chemically induced upregulation of Bcl-2 expression did not alter the increase in L444P GC activity mediated by UPR induction.

**Figure 7:**
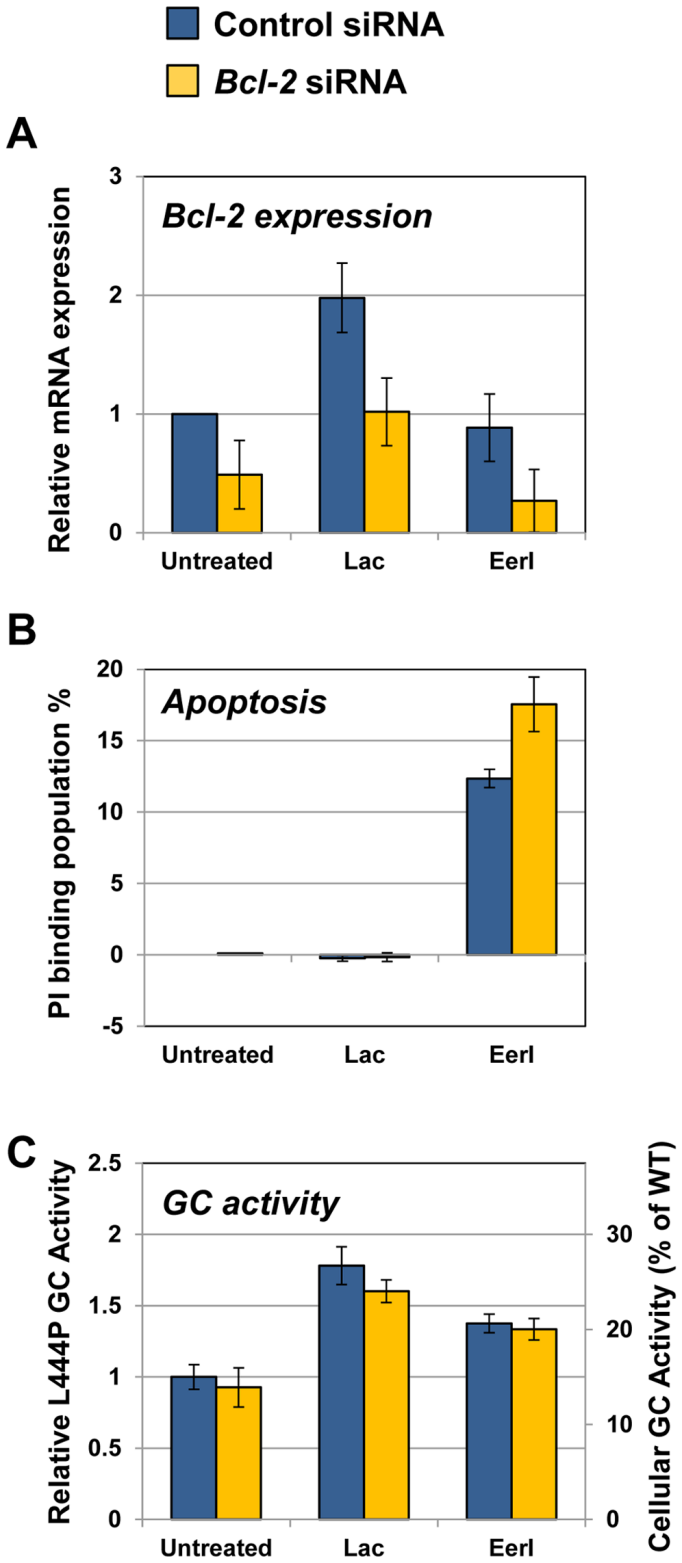
Silencing *Bcl-2* aggravates the apoptotic effect of proteostasis modulators. (**A**) Relative mRNA expression levels of Bcl-2 in GD fibroblasts incubated with siRNA for 48 hrs and treated with lacidipine (10 µM) and EerI (6 µM) for additional 24 hrs evaluated by quantitative RT-PCR and calculated as described in Figure 4 (ANOVA, p<0.05). (**B**) Flow cytometry analysis of PI binding population change (%) of GD fibroblasts incubated with siRNA for 48 hrs followed by lacidipine (10 µM) and EerI (6 µM) treatment for 16 hrs (ANOVA, p<0.01). The change in PI binding population (%) was calculated by subtracting PI binding values of cells treated with small molecules to that of cells only incubated with control siRNA. The data is reported as mean ± SD. Number of total counted cells: 10,000. (**C**) Relative L444P GC activities in cells incubated with Bcl-2 or control siRNA for 48 hrs followed by lacidipine (10 µM) and EerI (6 µM) treatment for additional 48 hrs. Relative GC activities were evaluated as described in Figure 1B (ANOVA, p<0.01). Experiments were repeated three times and data points are reported as mean ± SD. Lac, lacidipine.

To further investigate the role of Bcl-2 in GD cells cultured with proteostasis modulators, we asked whether the genetic modulation of Bcl-2 expression correlates with apoptosis induction in cells treated with EerI and lacidipine. To this end, we downregulated the expression of *Bcl-2* using small interfering RNA (siRNA) and evaluated apoptosis induction and GC activity. L444P GC fibroblasts were incubated with siRNA against endogenous Bcl-2 for 48 hrs followed by small molecule treatment (lacidipine 10 µM and EerI 6 µM) for additional 48 hrs (Figure 7). Non-targeting siRNA was used as a siRNA knockdown control. To determine the extent of silencing achieved, we first measured Bcl-2 expression by quantitative RT-PCR (Figure 7A). Bcl-2 siRNA resulted in 50% downregulation of Bcl-2 expression compared to non-targeting siRNA in untreated cells. Bcl-2 silencing was also observed to reduce Bcl-2 expression in cells treated with lacidipine or EerI, confirming that the knockdown of endogenous Bcl-2 was achieved in both untreated and small molecule-treated cells.

Administration of *Bcl-2* siRNA to achieve partial (50%) downregulation of its expression did not significantly change the induction of apoptosis (monitored by measuring PI binding as described above, Figure 3) in either untreated or lacidipine treated cells (Figure 7B). However, reducing the expression of Bcl-2 in EerI-treated cells resulted in significantly higher apoptosis induction leading to a 35% increase in cell death (Figure 7B). These data suggest that Bcl-2 plays a key role in preventing induction of apoptosis associated with sustained UPR activation.

To investigate whether silencing Bcl-2 expression influences the increase in L444P GC residual activity achieved with proteostasis modulators, we also tested GC activity in GD cells treated under the same conditions (Figure 7C). Not surprisingly, Bcl-2 siRNA did not significantly alter GC activity in cells treated with lacidipine and EerI, confirming that modulating the expression of Bcl-2 does not influence folding of mutated GC.

## Discussion

LSDs comprise a class of more than 50 inherited diseases. They are individually rare, but collectively represent one of the most prevalent genetic disorders in children [33], [34]. GD is the most common LSD (1 in 60,000 people) with the highest frequency in the Ashkenazi Jewish population (1 in 1,000) [35]. It presents highly variable clinical manifestations ranging from adult forms to acute or chronic infantile neuronopathic types [6]. Enzyme replacement therapy is currently available for most patients, but fails to treat several affected areas, particularly the skeleton and the brain [36]. Bone marrow transplantation can reverse non-neurological aspects of the disease, but it is rarely performed [37]. Inhibitors of glucosylceramide synthesis are available for the treatment of GD patients with moderate clinical manifestations for which enzyme replacement therapy cannot be considered an option [38]. In summary, there is no effective treatment for neurological symptoms that affect the brain damage that occurs in the most severe cases of GD.

The most prevalent mutations in gene encoding GC (*GBA*, NM_000157) result in single amino acid substitutions that do not directly impair enzymatic activity but destabilize the protein structure, leading to its degradation via ERAD [5]. These misfolding-prone GC variants, however, retain catalytic activity if forced to fold into their native structure [7]–[10]. In an effort to design therapeutic strategies that overcome the blood brain barrier and ameliorate symptoms in the central nervous systems, increasing focus has been recently devoted to the development of small molecule based strategies to rescue native folding of GC variants containing non-inactivating, destabilizing mutations and enhance their lysosomal targeting and activity. Particularly, modulating the proteostasis network to upregulate the synthesis, folding and processing of secretory proteins holds significant promise to efficiently rescue protein homoeostasis in GD cells [10]. A variety of small molecules including proteasome inhibitors [10], [39], Ca^2+^ blockers [13], [14], and ERAD inhibitors [15] were reported to enhance folding and activity of the most destabilized GC variant containing the L444P substitution, which is the most prevalent mutation in GD patients with CNS symptoms. However, the mechanism of action of these proteostasis modulators involves induction of ER stress and activation of the UPR. Sustained activation of the UPR, in turn, can lead to apoptosis thus ultimately compromising effective rescue of protein homeostasis. In this study, we sought to investigate chemical strategies to selectively modulate different branches of the proteostasis network that enhance rescue of L444P GC folding, while preventing proteotoxic stress and apoptosis.

We previously reported that inhibition of specific steps of ERAD leads to rescue of L444P GC folding and activity. As expected, ERAD inhibition enhances retention of unstable GC in the ER and ultimately results in enhanced lysosomal trafficking and activity, but it also leads to significant UPR induction and apoptosis [15]. On the other hand, restoring Ca^2+^ homeostasis via lacidipine treatment proved to be an effective strategy to modulate the cellular folding capacity and rescue L444P GC folding and activity without, however, inducing apoptosis [14]. In this study, we attempted to simultaneously increase retention of GC folding intermediates into the ER (via ERAD inhibition) and enhance ER folding (by restoring Ca^2+^ homeostasis). We found that combining these two mechanisms of proteostasis modulation enhances lysosomal trafficking and activity of L444P GC (Figure 1). Most importantly, we proved that the observed increase in GC activity was accompanied by lowered apoptosis induction, which suggests that lacidipine treatment protects GD cells from induction of UPR-associated apoptotic response.

Lacidipine treatment remodels the UPR pathway activated by EerI. Remodeling of the UPR, in turn, seems to be tightly linked to lacidipine’s anti-apoptotic function. Signal transducers of the UPR can activate either cytoprotective or pro-apoptotic pathways [29]. A pro-survival response is first initiated to reduce the load of misfolded proteins by boosting the ERAD pathway [40]. This response is mediated by the induction of the IRE1 signaling cascade [41] via Xbp-1 splicing and activation [28]. If the proteotoxic stress persists, pro-apoptotic signals are elicited through the activation of the PERK and ATF6 signaling cascades through the expression of ATF4 and its target CHOP. Induction of this pro-apoptotic response occurs simultaneously to attenuate IRE1 signaling [29], [42]. We found that lacidipine enhances EerI-mediated Xbp-1 splicing and lowers the activation of ATF4 and CHOP. These results suggest that lacidipine remodels EerI-mediated UPR induction by activating the anti-apoptotic IRE1 signaling cascade and inhibiting the activation of the pro-apoptotic PERK and ATF6 arms.

The Bcl-2 protein family plays a key role in the activation of UPR-associated apoptosis [43]. Particularly, Bcl-2 is an essential anti-apoptotic protein that controls cell survival [31] and is possibly involved in maintaining Ca^2+^ homeostasis by reducing [Ca^2+^]_ER_ efflux [44], [45]. Bcl-2 is upregulated upon treatment of GD fibroblasts with lacidipine [14]. We showed herein that lacidipine enhances the expression of Bcl-2 in EerI-treated cells and protects cells from EerI-mediated apoptosis induction. We also reported direct evidence that the expression level of Bcl-2 plays a protective role in GD cells treated with proteostasis modulators that activate the pro-apoptotic arms of the UPR.

In conclusion, this study demonstrates that combining modulation of Ca^2+^ homeostasis and ERAD inhibition enhances the ER folding capacity of GD fibroblasts thereby enabling efficient rescue of folding, trafficking and activity of L444P GC. Generally speaking, there is a clear correlation between protein stability and extent of degradation and residual enzymatic activity of GC variants containing destabilizing, non-inactivating mutations. Accordingly, modulation of the proteostasis network typically result in higher rescue of enzymatic activity in cells expressing highly destabilized GC variants, such as L444P GC, than in cells expressing less unstable variants that retain higher enzymatic activity, such as N370S GC [15]. The results reported herein provide insights for the development of pharmacologic strategies to modulate the proteostasis network and rescue native folding of unstable, degradation-prone proteins traversing the secretory pathway without triggering induction of ER stress and activation of apoptosis.

## Supporting Information

Figure S1 **Co-treatment of GD patient-derived fibroblasts with EerI and lacidipine enhances the folding, lysosomal trafficking and activity of L444P GC.** Relative L444P GC activities were evaluated in cells treated with a range of concentrations of EerI and constant doses of lacidipine (5, 10, or 20 µM) for 72 hrs. Relative GC activities were evaluated by normalizing GC activities measured in treated cells to the activity in untreated cells (left y axis). The corresponding fraction of WT GC activity is also reported (right y axis). Experiments were repeated three times and data points are reported as mean ± SD. Lac, lacidipine.
(DOCX)

Figure S2 **Chemically induced upregulation of Bcl-2 enhances mutated GC activity rescue.** Relative L444P GC activities of GD fibroblasts treated with EerI (2 and 6 µM), MG-132 (0.6 µM), and fluvastatin (100 nM) for 72 hrs. Relative GC activities were evaluated as described in Figure S1. Experiments were repeated three times and data points are reported as mean ± SD. MG, MG-132; Flu, fluvastatin.
(DOCX)

Table S1 **Primers.**
(DOCX)

File S1 (DOCX)
