# Supplementary figures and images for: Remodeling the Proteostasis Network to Rescue Glucocerebrosidase Variants by Inhibiting ER-Associated Degradation and Enhancing ER Folding
Source: PLoS One. 2013 Apr 19;8(4):e61418. doi: 10.1371/journal.pone.0061418 (PMC3631227; doi:10.1371/journal.pone.0061418)

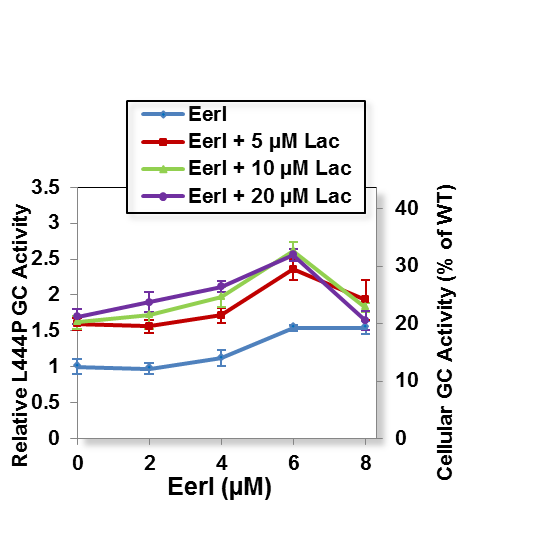

Supplement: Figure S1 — Co-treatment of GD patient-derived fibroblasts with EerI and lacidipine enhances the folding, lysosomal trafficking and activity of L444P GC. Relative L444P GC activities were evaluated in cells treated with a range of concentrations of EerI and constant doses of lacidipine (5, 10, or 20 µM) for 72 hrs. Relative GC activities were evaluated by normalizing GC activities measured in treated cells to the activity in untreated cells (left y axis). The corresponding fraction of WT GC activity is also reported (right y axis). Experiments were repeated three times and data points are reported as mean ± SD. Lac, lacidipine. (DOCX) [file pone.0061418.s001.docx]
