# Supplementary material for: Remodeling the Proteostasis Network to Rescue Glucocerebrosidase Variants by Inhibiting ER-Associated Degradation and Enhancing ER Folding
Source: PLoS One. 2013 Apr 19;8(4):e61418. doi: 10.1371/journal.pone.0061418 (PMC3631227; doi:10.1371/journal.pone.0061418)
Supplement: Figure S2 — Chemically induced upregulation of Bcl-2 enhances mutated GC activity rescue. Relative L444P GC activities of GD fibroblasts treated with EerI (2 and 6 µM), MG-132 (0.6 µM), and fluvastatin (100 nM) for 72 hrs. Relative GC activities were evaluated as described in Figure S1. Experiments were repeated three times and data points are reported as mean ± SD. MG, MG-132; Flu, fluvastatin. (DOCX) [file pone.0061418.s002.docx]

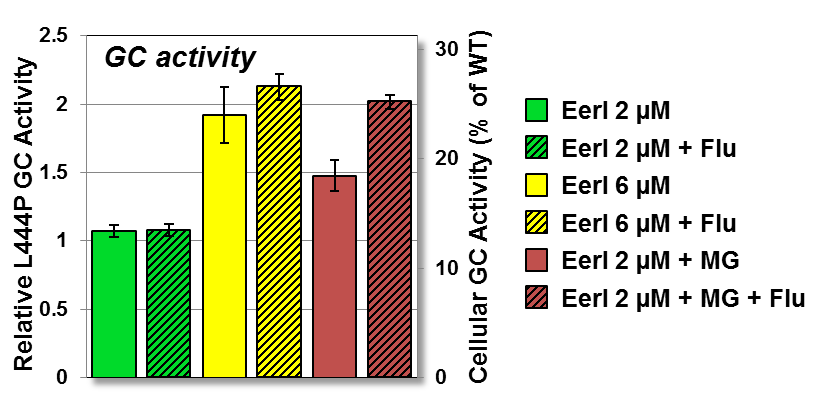


**Figure S2. Chemically induced upregulation of Bcl-2 enhances mutated GC activity rescue.** Relative L444P GC activities of GD fibroblasts treated with EerI (2 and 6 µM), MG-132 (0.6 µM), and fluvastatin (100 nM) for 72 hrs. Relative GC activities were evaluated as described in Figure S1. Experiments were repeated three times and data points are reported as mean ± SD. MG, MG-132; Flu, fluvastatin.
