# Supplementary material for: Remodeling the Proteostasis Network to Rescue Glucocerebrosidase Variants by Inhibiting ER-Associated Degradation and Enhancing ER Folding
Source: PLoS One. 2013 Apr 19;8(4):e61418. doi: 10.1371/journal.pone.0061418 (PMC3631227; doi:10.1371/journal.pone.0061418)
Supplement: Table S1 — Primers. (DOCX) [file pone.0061418.s003.docx]

| Gene | GenBank  Accession Code | Primer Name | Sequence |
| --- | --- | --- | --- |
| Xbp-1 | NM_005080 | Xbp1.f | 5'-TTA CGA GAG AAA ACT CAT GGC-3' |
|  |  | Xbp1.r | 5'-GGG TCC AAG TTG TCC AGA ATG C-3' |
| ATF4 | NM_182810 | ATF4.f | 5’-GAC CAC GTT GGA TGA CAC TTG-3’ |
|  |  | ATF4.r | 5’-GGG AAG AGG TTG TAA GAA GGT G-3’ |
| CHOP | NM_004083 | CHOP.f | 5'-ACC AAG GGA GAA CCA GGA AAC G-3' |
|  |  | CHOP.r | 5'-TCA CCA TTC GGT CAA TCA GAG C-3' |
| Bcl-2 | NM_000633 | Bcl-2.f | 5'-GGG GAG GAT TGT GGC CTT C-3' |
|  |  | Bcl-2.r | 5'-CAG GGC GAT GTT GTC CAC C-3' |
| GC | NM_000157 | GC.f | 5'-CCA AGC CTT TGA GTA GGG TAA G-3' |
|  |  | GC.r | 5'-CCC GTG TGA TTA GCC TGG AT-3' |
| GAPDH | NM_002046 | GAPDH.f | 5'-GTC GGA GTC AAC GGA TT-3' |
|  |  | GAPDH.r | 5'-AAG CTT CCC GTT CTC AG-3' |
