# Supplementary material for: Remodeling the Proteostasis Network to Rescue Glucocerebrosidase Variants by Inhibiting ER-Associated Degradation and Enhancing ER Folding
Source: PLoS One. 2013 Apr 19;8(4):e61418. doi: 10.1371/journal.pone.0061418 (PMC3631227; doi:10.1371/journal.pone.0061418)
Supplement: File S1 — (DOCX) [file pone.0061418.s004.docx]

**Remodeling the Proteostasis Network to Rescue Glucocerebrosidase Variants by Inhibiting**

**ER-associated Degradation and Enhancing ER Folding**

Fan Wang^1^ and Laura Segatori^1,2,3*^

Departments of ^1^Chemical and Biomolecular Engineering, ^2^Biochemistry and Cell Biology and ^3^Bioengineering, Rice University, CHBE-MS 362, 6100 Main St. Houston, TX 77005.

*Correspondence: Laura Segatori

E-mail: segatori@rice.edu

Phone: 713-348-3536 Fax: 713-348-5478

Rice University, CHBE-MS 362, 6100 Main St. Houston, TX 77005

**Supplementary material**

**Reagents and cell culture**

Eeyarestatin I was purchased from ChemBridge. Lacidipine and Conduritol B Epoxide (CBE) were from Toronto Research Chemicals. Fluvastatin was from Enzo Life Sciences. 4-methylumbelliferyl β-D-glucoside (MUG) was from Sigma-Aldrich. Cell culture media were from Lonza.

GD patient-derived fibroblasts homozygous for the L444P (1448T>C) mutation (GM10915) were obtained from Coriell Cell Repositories. Fibroblasts were grown at 37°C in 5% CO2 in minimal essential medium with Earle’s salts, supplemented with 10% heat-inactivated fetal bovine serum and 1% glutamine Pen-Strep. Medium was replaced every 3 or 4 days. Monolayers were passaged with TrypLE Express.

**Enzyme activity assays**

The intact cell glucocerebrosidase (GC) activity assay was performed as previously described [10]. Briefly, 100 μl aliquots of 104 cells were plated in each well of a 96-well plate and incubated overnight to allow cell attachment. The medium was replaced with fresh medium containing small molecules (small molecule concentrations and time of incubation are specified in each experiment) and plates were incubated at 37°C. The medium was then aspirated and cells were washed with PBS three times. The assay reaction was started by the addition of 50 μl of 2.5 mM 4-methylumbelliferyl β-D-glucoside (MUG) in 0.2 M acetate buffer (pH 4.0) and stopped after 7 hrs of incubation at 37°C by the addition of 150 μl of 0.2 M glycine buffer (pH 10.8) to each well. Liberated 4-methylumbelliferone was measured (excitation 365 nm, emission 445 nm) with a SpectraMax Gemini plate reader (Molecular Device). Non-lysosomal GC activity was evaluated by measuring GC activities in the presence of Conduritol B Epoxide (CBE) at 1 mM final concentration. Relative GC activities were calculated by subtracting the background of non lysosomal activity and normalizing the obtained values by the activity of untreated cells.

**Quantitative RT-PCR**

Quantitative RT-PCR was performed as previously described [15]. Cells were incubated with small molecules for 24 hrs before total RNA was extracted using RNAGEM™ reagent (ZyGEM). cDNA was synthesized from total RNA using qScript™ cDNA SuperMix (Quanta Biosciences). Total cDNA amount was measured by NanoDrop 2000 (Thermo Scientific). Quantitative PCR reactions were performed using cDNA, PerfeCTa™ SYBR Green FastMix™ (Quanta Biosciences) and corresponding primers (Table S1) in the CFX96™ Real-Time PCR detection system (Bio-Rad). Samples were heated for 2 min at 95°C and amplified in 45 cycles of 1 s at 95°C, 30 s at 60°C, and 30 s at 72°C. Analyses were conducted using CFX manager software (Bio-Rad) and the threshold cycle (C_T_) was extracted from the PCR amplification plot. The Δ C_T_ value was used to describe the difference between the C_T_ of a target gene and the CT of the housekeeping gene, GAPDH: ΔC_T_ = C_T_ (target gene) - C_T_ (GAPDH). The relative mRNA expression level of each target gene in treated cells was normalized to that measured in untreated cells: relative mRNA expression level = 2 exp [-(ΔC_T_ (treated cells) - ΔC_T_ (untreated cells))]. Each data point was evaluated in triplicate and measured three times.

RT-PCR analysis of Xbp-1 splicing was performed using total cDNA, Taq DNA polymerase and the Xbp-1 primers listed in Table S1 following reverse transcription. PCR products were separated on a 2.5% agarose gel. Spliced Xbp-1 bands were quantified by NIH ImageJ analysis software.

**Western blot analyses**

Cells were incubated with small molecules for 48 hrs, collected and lysated with the complete lysis-M buffer containing the protease inhibitor cocktail (Roche). Total protein concentrations were determined by Bradford assay (Thermo Scientific) and each sample was diluted to the same protein concentration. Aliquots of cell lysates were separated by 10% SDS-PAGE gel, and Western blot analyses were performed using primary antibodies (rabbit anti-BiP and mouse anti-CRT (Stressgen), mouse anti-CNX and rabbit anti-Glucocerebrosidase (Sigma-Aldrich), or rabbit anti-GAPDH (Santa Cruz Biotechnology)) and appropriate secondary antibodies (HRP-conjugated goat anti-rabbit (Santa Cruz Biotechnology) or goat anti-mouse IgG (Stressgen)). Blots were visualized using Luminata Forte Western HRP substrate (Millipore) and quantified by NIH ImageJ analysis software.

**Immunofluorescence studies**

Fibroblasts were seeded on glass coverslips, cultured in the presence of small molecules for 48 hrs, and fixed with 4% paraformaldehyde for 30 min. Cells were permeabilized with 0.1% Triton-X for 5 min and incubated with 8% BSA for 1 hr. Following 1 hr of incubation with primary antibodies (rabbit anti-β-glucocerebrosidase and mouse anti-CNX antibodies, Sigma-Aldrich), cells were washed three times with 0.1% Tween-20/PBS, and then incubated with secondary antibodies for 1 hr (Dylight 488 goat anti-mouse IgG and Dylight 549 goat anti-rabbit IgG from KPL, and FITC anti-LAMP-1 from Biolegend). Images were obtained using an Olympus IX81 confocal microscope and co-localized using the Fluoview software. Co-localization heatmap images were analyzed using NIH ImageJ analysis software.

**Toxicity assay**

L444P patient-derived cells were treated with EerI (6 μM) and lacidipine (10 μM) for 16 hrs at 37 ºC. Cells were collected and cell toxicity was tested with the CytoGLO^TM^ Annexin V-FITC Apoptosis Detection Kit (IMGENEX) according to the manufacturer’s instructions and analyzed by flow cytometry (FACSCanto^TM^ II, Beckon Dickingson) with a 488-nm Argon laser.
